# Trauma-informed care in geriatric inpatient units to improve staff skills and reduce patient distress: a co-designed study protocol

**DOI:** 10.1186/s12877-021-02441-1

**Published:** 2021-09-10

**Authors:** Monica Cations, Kate Laver, Leah Couzner, Stephen Flatman, Petra Bierer, Catherine Ames, Yan Huo, Craig Whitehead

**Affiliations:** 1 College of Education, Psychology and Social Work, Flinders University, GPO Box 2100, SA Adelaide, Australia; 2 South Australian Health and Medical Research Institute, Adelaide, SA Australia; 3 College of Medicine and Public Health, Flinders University, Adelaide, SA Australia; 4 Southern Adelaide Local Health Network, SA Health, Adelaide, SA Australia

**Keywords:** Inpatient geriatric care, Hospital care, Trauma-informed care, Mental health, Psychological wellbeing, Behavioural and psychological symptoms of dementia

## Abstract

**Background:**

Geriatric hospital wards are highly medicalised environments with limited opportunities for choice and control, and can be distressing for older survivors of psychological trauma. While trauma-informed models of care (TIC) are effectively applied across mental health and other settings, the utility of these models in aged care settings has not been assessed. The objective of this study was to examine whether TIC can reduce responsive behaviour, chemical restraint, and improve staff skills and patient experiences in inpatient geriatric settings.

**Methods:**

Four wards participated in this type I hybrid implementation-effectiveness study across southern Adelaide, Australia, including 79 beds. Using a co-design method, the principles of TIC were transformed into an implementation strategy including staff training, establishment of highly trained ‘champions’ on each ward, screening for trauma-related needs, and amending ward policies and procedures. Primary outcomes will be examined using an interrupted time-series design and are monthly incidence of responsive behaviour incidents and use of chemical restraint. Process evaluation will be used to examine secondary, implementation outcomes including the acceptability, feasibility, and fidelity to the implementation strategy.

**Discussion:**

Trauma-informed care has potential to improve the safety and accessibility of hospital wards for older people who have survived psychologically traumatic events and has an extensive evidence base supporting its effectiveness in other settings. Identifying trauma-related needs and amending care to reduce the risk of re-traumatisation and distress may also reduce the incidence of responsive behaviour change, which has a significant impact on the quality of life of hospital patients and staff and is very costly. The inclusion of a process evaluation will allow us to identify and report changes made on each ward and make recommendations for future implementation efforts.

## Background

Patient distress that results in responsive behaviours is a significant problem for geriatric inpatient units. Incidents are most common among people with dementia: seventy-five per cent will exhibit responsive behaviour during an inpatient stay, most commonly aggression (57 %) and other disturbance during personal care (44 %) [1]. Responsive behaviours are highly disruptive and distressing for the person, their families, other inpatients, and hospital staff. Older people who exhibit responsive behaviours in acute settings have increased mortality and are more likely to fall compared to those who do not [1]. Other inpatients report feeling anxious and afraid of aggressive and agitated patients [2] and are the victims of violence in 23 per cent of reported cases [3]. Responsive behaviour is also a key contributor to staff “burnout” and turnover [4, 5]. Staff consistently report lacking confidence and expertise to manage responsive behaviours [6].

Low skill and confidence in managing responsive behaviour may lead to an overreliance on chemical restraint [7]. Sedating medications including antipsychotics are commonly used to manage responsive behaviour in aged care settings [8]. The efficacy of these medications is low [9] and they are associated with serious side effects including increased mortality [10]. A recent longitudinal study identified that patients administered antipsychotic medications on geriatric inpatient wards were three-times more likely to die during their stay regardless of their health at admission [11].

Psychological trauma is a key contributor to responsive behaviour in health settings. Trauma can impair mood and behaviour regulation and this can make it difficult for survivors to cope with stressors (including triggers), interact effectively with other people, express their needs, and learn from their own and others’ experiences [12]. These difficulties can manifest in ways that are not always immediately obvious. Psychological trauma is well recognised as an important contributor to responsive behaviour in acute mental health settings [13].

The extent to which psychological trauma contributes to responsive behaviours in older people and people with dementia is unknown. However, up to 70 % of older people have experienced a psychologically traumatic event in their lives [14], and these experiences lead to clinically significant post-traumatic stress disorder in 20–40 % of cases [14]. Older people may have more difficulty coping with past trauma as their social supports fade and coping strategies become less effective [14]. Common aged care practices (e.g. assisting with toileting, bathing, and dressing) and environments (e.g. locked wards) can retrigger trauma reactions and be unsafe for survivors [15, 16]. This is particularly likely in the context of dementia given deficits in orientation to time and place, memory, cognitive inhibition, and communication. The loss of control implicit to receiving dementia care directly threatens the most important component of recovery from trauma [17, 18]. There is increasing consensus that many responsive behaviours exhibited by people with dementia occur in response to unmet needs [19]. However, no work has been done to determine whether addressing trauma-related needs can reduce behaviours and improve the wellbeing of people with dementia.

Trauma-informed care (TIC) is a model of care originally developed for psychiatric settings in recognition of the fundamental impacts that psychological trauma can have on perception and relationships and as a maintaining factor for other problems [20]. TIC was designed to be a systemic change approach in which a care setting is designed (or re-designed) to promote the three core components of trauma recovery: restoring power (via maximising choice, autonomy, and control), creating safety, and building patient and staff self-worth [20]. Trauma-informed care extends the philosophy of person-centred care by emphasising the fundamental role of trauma in shaping the person’s experience of care [18]. It is also distinct from trauma-focussed therapies or services; a trauma-informed setting is not required to treat the symptoms of the trauma but rather ensure that all staff members are able to incorporate their knowledge of trauma, its impact, and referral pathways into their daily practice [21].

There have been many efforts to translate trauma-informed models of care into health care settings including emergency departments [22] and hospice care [23] using organisation-level interventions. However, to our knowledge there have been no studies aiming to evaluate implementation of TIC into acute or subacute aged care settings. Implementing trauma-informed care into acute geriatric settings has several potential benefits. Building a sense of trust and control helps trauma survivors cope with unpleasant situations, improving quality of life and quality of care [21]. Preventing responsive behaviour or effectively using non-pharmacological strategies to respond reduces staff burden, distress for other patients, and the need for chemical restraint. As antipsychotic medications prescribed in acute settings are less likely to be reviewed and discontinued than those prescribed in the community [24], limiting their use in this setting can have ongoing benefits for the person after discharge.

This study will assess the utility of trauma-informed care in generalist acute geriatric units. It will identify the impact of addressing trauma-related needs on responsive behaviour, chemical restraint, and staff skills. It will establish barriers and facilitators to implementation in acute geriatric contexts and guidelines for wider implementation.

### Objectives

The primary aim of this project is to evaluate the effect of implementing trauma-informed care in terms of reducing distress and associated responsive behaviours among patients in geriatric hospital wards. The secondary aim of this project is to examine the implementation approach, to understand barriers and facilitating factors for implementation and sustainability.

Research questions include:

- Can a trauma-informed organisational implementation strategy reduce responsive behaviour, chemical restraint, and improve staff skills in geriatric inpatient settings?
- Are the effects comparable across ward types / populations?
- What is the acceptability, feasibility, and cost of implementing a trauma-informed care organisational intervention?
- What are the barriers and facilitators for implementation success?

## Methods

### Design

We will assess the effectiveness and implementation of this multi-component trauma-informed organisational implementation strategy using an interrupted time-series design with inbuilt process evaluation over 36 months, meeting criteria for Curran’s Type I hybrid implementation-effectiveness design [25]. Interrupted time series will be used to monitor long term trends to estimate the impact of the implementation strategy. It is an ideal design where outcome data is routinely collected [26]. Mixed-methods process evaluation will be used to assess the implementation of TIC including its adoption, acceptability, and sustainability.

### Setting and participants

Older people account for a large proportion of the hospitalised population in Australia, but the organisation of acute care to meet their needs varies between regions. This study will take place in four hospital wards (within one health network) located across three public hospitals in the Southern Adelaide region of South Australia, Australia. Just over 18 per cent of the region’s population is aged over 65 years, equating to approximately 64,000 people [27].

Participating wards include two Geriatric Evaluation and Management Units that provide care to older people with and without dementia with multidisciplinary input to minimise disability and reduce the risk of placement in permanent residential aged care. The wards provide longer periods of rehabilitation and restorative care and so are distinct from other hospital wards offering short term acute health care [28]. There are 51 beds across these two wards serviced by medicine, nursing, physiotherapy, occupational therapy, speech pathology, dietetics, and social work professionals. Ward rooms are either single or shared by two to four patients, and each ward has a separate gym area for rehabilitation activities.

A third participating ward is a specialised 12-bed unit for people with dementia with high-level behavioural support needs. This unit was specifically designed to reduce the incidence of responsive behaviours with several private spaces and ability to separate the ward into two ‘pods’ to minimise risk of violence between patients. The final participating ward is a Transition Care Ward co-delivered by the public health service and an aged care provider. This ward delivers a hospital avoidance program allowing short term (up to 12 weeks) restorative care to older people leaving hospital (often other wards included here) to promote independence and transition to home.

These four wards were chosen for inclusion because they share common senior leadership including rotating specialist physicians, and because they service a diverse population of older adults from those without dementia or with mild impairments transitioning to home through to those with severe impairments and associated behavioural and psychological symptoms unable to be cared for in generalist residential aged care. Patients commonly transfer between these wards as their care needs change. In total there are 79 beds across these four wards that are consistently above 99 % occupancy. The average length of stay across the wards is 25 days with 19 % of patients transitioning or returning to residential aged care.

Participants in this project will include multidisciplinary staff working in the participating geriatric inpatient units and patients admitted for at least one night in the participating geriatric inpatient units. Staff will be involved in education, organisational change, and research data collection. Patients will receive changes to care protocols because of the implementation strategy, but all patient-related outcomes will be routinely collected by ward staff. Therefore, patients will not have direct contact with the research team.

### Co-design

A two-step process is recommended for implementation of TIC because it is necessary to transform the core principles into concrete actions and behaviours. Implementation guidelines recommend that this occurs via a co-design process with organisation management, staff, and advocacy groups to ensure that the behaviours are relevant and feasible for the given context [29, 30]. As such, a two-step implementation strategy development process was followed for this study. Three co-design meetings were held between September and December 2020 including 22 ward management, staff, consumer advocates, and researchers in total. Meetings were structured to gather staff views about how TIC could be operationalised in their setting, and included examples from previous studies (for example, the Six Core Strategies for Reduction of Seclusion and Restraint [31]). The meeting transcripts were thematically analysed to generate themes to guide development of the implementation strategy (Table 1) [32].

**Table 1:** Overview of themes generated from co-design meetings

Theme	Description
*Sense of relevance*	Most ward staff could reflect on previous or current patients with complex needs hypothesised or confirmed to be related to psychological trauma
*Behaviour management focus with lack of adequate preparation*	A clinical focus on behaviour management among staff was often used to frame understanding of trauma-informed care; framing implementation strategy through this paradigm will maximise acceptability and buy-in
*Low mental health expertise*	Need to improve psychological literacy across all ward staff
*Limitations to information sharing*	Room for improvement in how information about a patient’s history, triggers, and needs is shared

Most ward staff who participated in the meetings could reflect on experiences with previous or current patients with a hypothesised or confirmed trauma history, for whom providing care was challenging. From this perspective the participants endorsed the relevance and acceptability of the concepts, even where they lacked confidence in their ability to respond appropriately. This limited confidence was often related to organisational constraints on their ability to provide flexible care. The need for high-level organisational policy and procedure change to support implementation was emphasised.

During discussions, staff often returned to a behaviour management paradigm to make sense of the concepts of TIC. A significant shift in patient presentation in the participating wards had occurred in the years preceding co-design, with more frequent admission of people with dementia and responsive behaviour change. This had been a difficult transition for staff especially in wards that were not originally designed for this purpose. Staff felt that they were not generally well prepared for such complexity when entering the wards and welcomed efforts to improve capacity. Therefore the proposed TIC intervention was largely perceived as an opportunity to improve behaviour management skills. Tailoring the implementation strategy and language to fit within a behaviour management paradigm was required to maximise acceptability and buy-in.

Participants noted the absence of psychology or mental health nursing professionals on the participating wards and highlighted this as a potential barrier to TIC implementation. A team of social workers are available but the co-design group noted an opportunity for the mental health expertise of this team to be strengthened and better utilised. Overall, the need to improve the psychological literacy of all staff on the ward was considered necessary to ensure implementation success.

A fourth major theme generated from the co-design meeting transcripts concerned information sharing within and between ward staff. Though the wards are governed by the same management team, the co-design group noted room for improvement in the ways in which information is shared. Several participants noted that information about a patient’s history, triggers, and needs is not routinely shared (or accessed) from triage, admission assessments, and at discharge. However, vertical information sharing was noted as a strength on the wards as floor staff are given regular opportunities to share information with clinical leadership and management. This strength can be leveraged in the current project.

### Implementation strategy

From the co-design process, additional consultation with consumer advocates, and based on guidelines by Proctor et al. [33], a multi-component implementation strategy was designed and agreed by all stakeholders (Table 2). It involves elements that are common to most trauma-informed organisational interventions including work to build buy-in for TIC, establishing organisational protocols consistent with the principles of TIC, staff education, selection and upskilling of TIC ‘Champions’ using a learning collaborative model, and amending assessment procedures.

**Table 2:** Overview of implementation strategy

Implementation strategy	Description
Plan	
Gather information	∙ Co-design process ∙ Literature review to identify barriers and enablers to implementing trauma-informed care in health settings ∙ In-depth interviews with a selection of ward staff
Select strategies	∙ Develop implementation strategy components based on co-design process and literature review ∙ Implementation strategy updated
Build buy-in	∙ Presentations to ward staff to introduce initiative and provide rationale ∙ Establish steering group including research team, ward leadership, ward clinicians, and patient advocates ∙ Bi-monthly steering group meetings ∙ Internal media to promote the initiative
Initiate leadership	∙ Steering group includes ward leadership ∙ Champions identified as leaders within the wards
Educate	
Develop materials	∙ Design and produce online training module together with ward staff and educational design experts ∙ Update assessment forms to include screening for trauma-related needs ∙ Develop social work follow up protocol together with social work team
Educate	∙ Provision of online training module ∙ Provision of in-service trainings to complement online module ∙ Provision of advanced Champion training ∙ Champions engage in regular supervision with expert psychologist, are provided with resources and professional development opportunities
Educate through peers	∙ Champions build a Learning Collaborative to enhance peer-to-peer learning ∙ Champions diffuse their knowledge via a train-the-trainer approach
Restructure	
	∙ Organisational procedure developed to inform trauma-informed care processes ∙ Intake assessment forms amended to include screening for history of psychological trauma ∙ Protocol for social work follow up developed and implemented together with social work team ∙ Behaviour management plans and discharge plans updated to include trauma-related triggers and needs
Finance	
	∙ Training provided free of charge with continuing availability after project closure ∙ $750 stipend incentive for Champions for further professional development
Quality management	
	∙ Ongoing peer and expert supervision for Champions ∙ Reminders ∙ Monthly quality audits for fidelity checking ∙ Initiative promotion via internal and external media

#### Plan

A collaborative approach to implementation will be taken between the research team and ward staff by enacting and demonstrating leadership support for the initiative. A steering group will be established to lead and oversee all project components. The steering group will include researchers, ward leadership, clinical staff, and patient advocates, and will meet bi-monthly to build buy-in, report progress, identify necessary changes to the implementation strategy, and promote sustainability. A group of clinical ‘Champions’ will be recruited via an expression of interest and will be established as program leaders using internal media. Champions will include at least two clinical staff per ward, as well as at least one rotating specialist medical consultant. In addition to the co-design process described above, detailed qualitative interviews with a selection of ward staff will be conducted to establish potential barriers to implementation and readiness for change.

#### Educate

All ward staff will complete an online training module outlining the principles of TIC, approaches to assessing for a trauma history, identifying signs of distress, situational awareness, de-escalation strategies, and debriefing and information sharing. The e-module will be co-designed by the research team, ward staff, and experts in educational design and will take a case-based learning approach [34]. Online training will be complemented by in-service events in each ward to reinforce the training messages and answer specific questions. In-services will occur once every 6 months (for a total of three times) in each ward over the intervention period. In addition, Champions will complete an in-person 2-day training program aiming to build a more advanced understanding of the effects of psychological trauma and foster higher-level skills in managing trauma-related needs in health settings. Champions will further develop this knowledge using a learning collaborative approach in which they will receive regular peer and expert supervision with a psychologist with expertise in trauma-informed care, communicate via a closed online discussion group, and are presented with resources and professional development opportunities by their supervisor. Champions will be expected to diffuse their knowledge and skills using a train-the-trainer approach with at least two colleagues working on the same ward. Promotion of the TIC initiative will be conducted via media both within and outside the wards.

#### Restructure

An organisational procedure will be developed and implemented by the steering group to inform how trauma-related needs will be identified and met across all wards. Screening for a history of psychological trauma will be included at admission and conducted by experienced triage nurses. Positive screening will trigger referral to the social work team who will follow up and identify trauma-related needs according to a protocol to be developed by the social work team with support from the steering group. Existing behaviour management plans, used to inform staff of potential triggers for responsive behaviour and strategies to prevent these triggers, will be updated to include trauma-related triggers and needs. These details will also be added to existing discharge plans to facilitate information sharing between wards, with family carers, and with the residential care facility to which the patient is being discharged (where appropriate and relevant).

#### Finance

Training will be provided to staff and Champions free of charge, but time to complete the training and engage in the learning collaborative will be provided in-kind by ward management. The online training program will be available at no cost for ward staff for continued use after the project is finalised. Champions will have access to a $750 stipend during the implementation period to spend on further professional development activities.

#### Quality management

Quality audits will be conducted each month to assess fidelity (e.g. frequency of screening and follow up protocol) and this data will be presented to the steering group to determine amendments required to the implementation strategy. The learning collaborative will provide both peer and expert supervision for the Champions to continuously build their skills.

### Outcomes

The primary outcomes for this study are (a) incident reports related to responsive behaviour, and (b) the use of *pro ne rata* (PRN) chemical sedation in response to a responsive behaviour event. Existing, routinely collected and de-identified ward data will be used to collect these outcomes. Staff routinely report and rate severity of any patient behaviour that poses perceived or actual threat to people, property, or self. A ‘Code Black’ is the highest risk rating and is called when security attendance is required. Staff are also required to report when a PRN (i.e. as needed) psychotropic medication is administered in response to a responsive behaviour event. The incidence of each outcome per occupied bed-days per month will be calculated and monitored over time.

Process outcomes will be collected to assess the success of implementation according to the RE-AIM framework of knowledge translation (Table 3) [35]. Field notes will be used to track reach, which for this study will refer to the proportion of staff who complete the online training module within the intervention period, the proportion of staff who volunteer for Champion roles, the proportion of patients who are screened for trauma-related needs, and the proportion of those patients who are followed up according to the new protocol. Field notes will be supplemented with quality audits conducted once a month by a member of the research team. Audits will include review of clinical notes to identify evidence of screening, follow up, and care provision in response to trauma-related needs. Any other field notes related to project acceptability, feasibility, and sustainability will be retained and analysed, including contact between ward staff and the research team, information about staff turnover, adverse events, and other ward quality improvement initiatives that may affect implementation.

**Table 3:** Process outcomes

Domain	Outcome	Measure	When?
Reach	Uptake of education, uptake of Champion roles, fidelity to screening procedure, fidelity to follow up procedure	Field notes	Throughout project
Effectiveness	Knowledge, skills, and confidence in assessing trauma history and adapting care	In-depth qualitative interviews with staff Knowledge, Attitudes, and Practice Related to Trauma-Informed Practice questionnaire completed by staff	Prior to implementation period and end of implementation period
Adoption	Acceptability	In-depth qualitative interviews with staff	Prior to implementation period and end of implementation period
	Feasibility		
	Penetration		
Implementation	Fidelity to intervention	Field notes, quality audits	Throughout project
	Barriers and facilitating factors for implementation	Field notes In-depth qualitative interviews with staff	Throughout project Prior to implementation period and end of implementation period
	Adverse events	Field notes, quality audits	Throughout project
Maintenance	Fidelity to intervention over time	Field notes, quality audits	Throughout project
	Perceived sustainability	In-depth qualitative interviews with staff	End of intervention period

In-depth interviews with a selection of ward staff early in the project will establish expectations, readiness for change, perceived acceptability and feasibility of the implementation strategy, potential barriers to implementation, and refinements to promote implementation success. Interviews will be repeated at the end of the 18 months to understand staff experiences with the strategy, impacts on their practice, and barriers to implementation that were experienced. The Consolidated Framework for Implementation Research was used to design the interview questions to ensure that we capture the breadth of constructs known to affect implementation success [36, 37].

Finally, staff will complete a survey before implementation commences and again at the end of the implementation period assessing their understanding of trauma-informed care and their skills in responding to trauma-related needs. The 21-item ‘Knowledge Attitudes, and Practice Related to Trauma-Informed Practice’ tool [38] asks respondents to indicate the extent to which they agree with statements (from 1 = strongly disagree to 5 = strongly agree) examining knowledge about TIC (e.g. “Trauma can have lifelong effects that may span generations”), attitudes toward TIC (e.g. “Trauma-informed care is essential to working with older adults”), and trauma-related practice (e.g. “I maintain transparency in all interactions with patients”). The strong internal consistency of the measure has been validated (Cronbach α = 0.74–0.86) [38].

### Analysis

The impact of the intervention on the incidence of responsive behaviour reports and use of chemical restraint will be evaluated with a segmented regression analysis across distinct segments of time (in this case, before and after the roll out of trauma-informed care) [39]. The statistical power of segmented regression analysis is related to the estimated number of time points at which data will be recorded [40]. This study incorporates 36 months of routine data collection including an 18-month pre-intervention period (January 2020-July 2021) and an 18-month post-intervention period (November 2021-April 2023). A 3-month washout period will be applied (August-October 2021) as implementation strategy elements are rolled out across the wards. This trial is powered at 81 % to detect a change of minimum ± 25 monthly responsive behaviour reports (estimated effect size = 1, autocorrelation = 0.3, and α = 0.05) [40].

Qualitative interview and field note data will be transcribed verbatim. A combination of inductive and deductive thematic analysis will be used to identify themes related to acceptability and feasibility of the intervention and key barriers to implementation [32]. Using a combined approach will allow us to capture themes generated inductively while also examining those we sought a priori according to the CFIR framework [36, 37, 41]. Data elicited from field notes and records will be reported descriptively to establish the reach, fidelity, acceptability, and sustainability of the implementation strategy. Data from the Knowledge Attitudes, and Practice Related to Trauma-Informed Practice tool regarding TIC skills and practice will be presented descriptively, and mean scores at the beginning and end of the intervention will be compared using a t-test. The mixed data sources will allow for comparison of outcomes by wards, strategy fidelity, number of staff per ward who completed the training program, and other factors that may have affected implementation success.

## Discussion

To our knowledge, this is the first research study aiming to examine the impact of TIC in geriatric inpatient care settings. Trauma-informed care has potential to improve the safety and accessibility of hospital wards for older people who have survived psychologically traumatic events, and has an extensive evidence base supporting its effectiveness in other settings [12]. Identifying trauma-related needs and amending care to reduce the risk of re-traumatisation and distress may also reduce the incidence of responsive behaviour change, which has a significant impact on the quality of life of hospital patients and staff [1–4] and is very costly [42].

This study benefits from several strengths. Use of a co-design process ensures that the implementation strategy components are relevant, acceptable, and feasible to those implementing them (e.g. staff) and those receiving them (e.g. patients) [43]. The principles of TIC are well established [29] and several organisational interventions have been successfully implemented in other settings [12, 21]. This allows us to learn from past experiences when adapting principles for our setting. The inclusion of several wards that service a diversity of patients allows for examination of where implementation of TIC is most successful and why. Using existing, routinely-collected measures ensures that outcomes are reported in standardised ways that are not vulnerable to reporting bias, and increases efficiency.

There are nonetheless important limitations to this study. We have not included control sites as it was considered unethical to withhold the implementation strategy given its effectiveness in other settings. There was also high risk of contamination as the wards share a management team and staff commonly rotate between wards. Without a control group we are unable to account for the effects of initiatives or other events occurring simultaneously to our implementation strategy. Nonetheless, the interrupted time-series design will allow us to monitor change while accounting for trends that were occurring before the implementation strategy was rolled out. We will also collect and report data regarding other initiatives that may have affected our outcomes. Another important limitation is that the effects of trauma-informed organisational interventions can be slow to emerge, particularly in health settings [21]. Successful implementation of TIC relies on a cultural change within the organisation and, like other quality improvement initiatives, can be slowed by staff turnover, competing priorities, and other contextual factors [36]. As such, the 18-month follow up period may be insufficient to detect a significant effect. The inclusion of a process evaluation will allow us to identify and report changes made on each ward and make recommendations for future implementation efforts.

## Data Availability

Not applicable.

## Abbreviations

CFIR: Consolidated Framework for Implementation Research
PRN: *pro ne rata*
SADU: Specialist Advanced Dementia Unit
TCP: Transition Care Program
TIC: Trauma-informed care
